# UV-B light and its application potential to reduce disease and pest incidence in crops

**DOI:** 10.1038/s41438-021-00629-5

**Published:** 2021-09-01

**Authors:** Prisca Meyer, Bram Van de Poel, Barbara De Coninck

**Affiliations:** Division of Crop Biotechnics, Department of Biosystems, KU Leuven, 3001 Leuven, Belgium

**Keywords:** Plant immunity, Light stress

## Abstract

Ultraviolet-B radiation (280–315 nm), perceived by the plant photoreceptor UVR8, is a key environmental signal that influences plant growth and development and can reduce disease and pest incidence. The positive effect of UV-B on disease resistance and incidence in various plant species supports the implementation of supplemental UV-B radiation in sustainable crop production. However, despite many studies focusing on UV-B light, there is no consensus on the best mode of application. This review aims to analyze, evaluate, and organize the different application strategies of UV-B radiation in crop production with a focus on disease resistance. We summarize the physiological effects of UV-B light on plants and discuss how plants perceive and transduce UV-B light by the UVR8 photoreceptor as well as how this perception alters plant specialized metabolite production. Next, we bring together conclusions of various studies with respect to different UV-B application methods to improve plant resistance. In general, supplemental UV-B light has a positive effect on disease resistance in many plant–pathogen combinations, mainly through the induction of the production of specialized metabolites. However, many variables (UV-B light source, plant species, dose and intensity, timing during the day, duration, background light, etc.) make it difficult to compare and draw general conclusions. We compiled the information of recent studies on UV-B light applications, including e.g., details on the UV-B light source, experimental set-up, calculated UV-B light dose, intensity, and duration. This review provides practical insights and facilitates future research on UV-B radiation as a promising tool to reduce disease and pest incidence.

## Introduction: light and plants

Plants harvest solar radiation in the 400–700 nm spectrum (photosynthetically active radiation, PAR) and use this energy mainly for photosynthesis, while light between 280 and 730 nm induces photomorphogenic responses and can regulate complex growth and developmental processes. Plants not only sense light duration (quantity), timing, and intensity but they are also able to sense light quality (spectral composition) with specific photoreceptors that have different absorption and action spectra. As such, plants can monitor minor changes in their light environment and rapidly initiate responses to adapt their physiology. These features are now exploited in horticultural applications to enhance specific traits using supplemental artificial lighting^1,2^. The introduction of light-emitting diodes (LEDs) in crop production systems facilitates fine-tuning of specific light conditions but we require a better understanding of how light can modulate plant responses.

Light within the PAR spectrum, especially red (630–700 nm), blue (450–490 nm), and green (490–570 nm) light, but also outside (far-red >700 nm) the PAR spectrum has been implemented in horticultural crop production to steer plant biomass, accumulation of specialized metabolites, fruit quality, and plant morphology^2–12^. Also, UV light (10–400 nm), which has been intensively investigated but not yet generally implemented in crop production systems, appears to have a broad effect on general plant growth and development. It influences e.g., the biosynthesis of specialized metabolites, flowering time, plant biomass, and plant architecture (branching and compactness)^13^. Moreover, UV light has been demonstrated to directly harm plant pathogens and increase disease resistance in plants^14,15^.

UV light is divided into UV-A (400–315 nm), UV-B (315–280 nm), and UV-C (280–10 nm) radiation. Only 7% of the solar radiation consists of UV light and the actual amount and spectrum of UV radiation reaching the earth’s surface is depending on the composition of the ozone (O_3_) layer. Typically, no UV-C light reaches the earth’s surface, while UV-B radiation is partly absorbed by atmospheric gases in the stratosphere and UV-A radiation completely passes through the ozone layer^16,17^. Both UV-B and UV-C light have been applied in horticulture to prevent disease outbreaks, however via a different mode of action. Supplemental UV-C light can reduce disease incidence in crops and is often applied to diminish postharvest decay of plant products or as a pre- and postharvest disinfection tool in horticulture^14,18–21^. The beneficial effect of UV-C light is mainly attributed to its direct genotoxic effect on plants and pathogens^21,22^. UV-B radiation on the other hand has a dual function. UV-B light can induce direct DNA and protein damage (UV-B light stress) and it also activates the UV-B photoreceptor UVR8 (UV-B RESISTANCE 8), which leads to a change in expression of various genes involved in UV-light stress acclimation^13^. While no UV-B photoreceptor has been identified in microorganisms yet, it is highly conserved among the plant kingdom and has even been found in mosses and algae (reviewed in Tilbrook et al. ^23^ and Vanhaelewyn et al.^24^).

Several studies focused on the use of supplemental UV-B light as a tool to increase disease resistance in various crops, but a lot of variation in the experimental setups make it challenging to compare the effect of UV-B radiation and draw general conclusions. However, in nearly all studies, a positive effect was observed. UV-B light can be applied at different (1) intensities, (2) durations (reaching from seconds to hours), (3) doses (dose = intensity × duration), (4) timing (e.g., in the morning, noon, afternoon, or night), and (5) infection stages (UV-B treatment before, during or after pathogen/pest inoculation). In addition, variation arises due to different crops used, environmental conditions (in vitro, greenhouse, open field, or indoor growth room setups), and UV-B light sources (narrowband, broadband, solar UV-B, filtered solar UV-B). It has even been shown that the sex of *Populus cathayana* determines how these plants react to UV-B light^25^. In order to provide an overview and facilitate further research in UV-B, we calculated actual UV-B values (dose, intensity, and duration) for many recent studies and highlighted their main effects (Table 1). Optimizing supplemental UV-B radiation holds the potential to reduce disease incidence and thus improve crop production and quality. However, despite many studies focusing on UV-B radiation, the overall consensus on the application method of UV-B light is not yet clear. This review aims to summarize and unravel the application potential of UV-B radiation in crop production with a focus on reducing disease and pest incidence.

**Table 1:** Studies using UV-B light as a tool to increase disease resistance in crops

Crop	Pathogen	Application time	Light set-up^a^	UV-B source	Daily dose, kJ m^−2^	Intensity, W m^−2^	Duration	Disease resistance^b^	Specialized metabolites, hormones, proteins	Source
Cucumber	*Podosphaera xanthii*	Night	G; UV-B in the background or R, B (39 µmol m^−2^ s^−1^ or UV-A (15.84 kJ kJ m^−2^; 2.2 W m^−2^)	Broad spectrum (Model UV-B-313EL: Q-PANEL Lab Products); ~280–380 nm, peak at 313 nm,	0.3–0.9	1	5, 10, 15 min	+	- Flavonols - Anthocyanins, Both after inoculation with pathogen	^122^
Rose	*Podosphaera pannosa*	Night or day	G	Broad spectrum; 265–385 nm, peak 312 nm	0.5–3	0.065–0.14 (average: 0.102)	2 h (night), 4 h, 6 h	+ for 2 h night and 6 h day treatment;4 h day treatment depending on cultivar		^33^
Rice	*Magnaporthe oryzae*	Day 10–17 h	Rice terrace	Broad-spectrum 40 W UV-B lamp 280–315 nm	2.5; 5; 7.5	0.099; 0.198; 0.297	7 h, Lower doses showed a better effect	+	+ Flavonoids (bell-shaped UV dose response), + phenols (dose-dependent) + PAL, LOX at higher doses + ß-1,3-glucanase increase with dose	^113^
Tobacco	*Spodoptera litura*	Day (pulsed)	G	Broadband (Philips TL 40 W/12) 290–315 nm	3	0.318	3 × 52.4 min (=157.2 min)	+	+ JA and JA-Ile + chlorogenic acid + rutin + dicaffeoylspermidine	^65^
Rice	*Mythimna separata, Chilo suppressalis*							+	+ JA and JA-Ile +/− p-coumaroylputrescine +/− feruloylputrescine +/− caffeoylputrescine	
Maize	*Spodoptera litura, Mythimna separata*							+	+ JA and JA-Ile +/− DIMBOA-Glc + HDMBOA-Glc	
Arabidopsis	*Spodoptera litura*	Day (pulsed)	L; 150 µmol/m^2^/s WL (Philips Essential TL5 28 W/865)	Broadband (Philips TL 40 W/12) 290–315 nm	1	0.318	3 × 17.4 min (=52.4 min)	+	+ JA and JA-Ile	
Strawberry	*Podosphaera aphanis*	Day + night	G	Broad spectrum (Model UV-B-313EL: Q-PANEL Lab Products); ~280–380 nm, peak at 313 nm, included UV-A radiation	0.288; 0.864	0.8; 1.6	18 min/every 3rd day, 6 min/daily 3 × 2 min/daily	+		^168^
Rosemary	*Golovinomyces biocellatus*							+		^168^
Rice	*Magnaporthe oryzae*	Day	G	40 W UV-B lamps (280–320 nm)	5	0.198	7 h	+	+ LOX, PAL, CHT activity + ß-1,3-glucanase + flavonoids + phenolics	^114^
Strawberry	*Sphaerotheca aphanis*	Day	L	n.m.	5–32	n.m.	n.m.	+ 5 kJ m^−2^ reduced disease level to 0		^101^
			Vinyl house		2–7			+		
			L		10				+ *FaCHi2–1* expression	
Strawberry	*Podosphaera aphanis*	Day	Vinyl house	n.m.	1.6–6.4	0.044–0.178	10 h	+		^160^
Strawberry	*Podosphaera aphanis*				0.2–6.5	0.018–0.602	3 h	+		
Strawberry	*–*		Experimental booth, WL		5.4	0.15	10 h		+/− GAPDH + ß-1,3-glucanase + osmotin like protein +CHI1 +PAL, CHS, CHI	
Arabidopsis	*Botrytis cinerea*	Day	L	UV-B TL100W/01, Phillips + clear polyethylene film (305–320 nm)	5.5	0.382	4 h	+	+sinapates	^64^
Arabidopsis	*Pieris rapae, Trichoplusia ni*	Day	L, WL	Phillips, HPA-400	1.5	0.0258	16 h	+ *P. rapae* +/− *T. ni*	+flavonoids	^173^
Soybean	*Nezara viridula, Piezodorus guildinii*	Day	Field	Solar control: UV-B blocking polyester films (cutoff below 320 nm)	n.m.	n.m.	n.m.	+	+ isoflavonoids daizin and genistin	^119^
Soybean	“typical soybean pests”: thrips, sting bugs, grasshoppers	Day	Field	Solar control: UV-B blocking clear polyester (cutoff below 320 nm)	n.m.	n.m.	n.m.	+		^174^
Soybean	*Spodoptera frugiperda*	Day	Field	Solar control: UV-B blocking polyester foil (cutoff below 380 nm)	n.m.	n.m.	n.m.	Herbivore did not differentiate between UV-B primed or not primed leaves	+faster phenolic accumulation + isorhamnetin- & quercetin-based flavonoids	^120^
Arabidopsis	*Spodoptera littoralis, Myzus persicae*	Day	L, WL	Broadband 290–315 nm TL12 tubular UV-B lamps (Philips) (filtered)	10	0.7	4 h	+	+ flavonoid type pigments	^121^
Broccoli	*Myzus persicae, Pieris brassicae*	Day	L, WL	Broadband (280–360 nm) Philips L40W/12 RS	1	0.056	5 h	+	+ total aliphatic glucosinolates (day 1 and 5) +indolyl glucosinolates (day 3) 3 days past treatment: + aliphatic glucosinolates, compositional change of indolyl glucosinolates Composition change in indolyl glucosinolates	^141^
	–	Day	L, WL	Broadband (280–360 nm) Philips TL20W/12 RS	0.6	0.042	4 h	n.a.	+ flavonoids (esp. kaempferol) + individual (+total) aliphatic glucosinolates -*CHS, -FLS* *+ CHI, PBS3* *+ PR-1, PR-2, PR-4, BG3, osmotin34*	
	–				2 × 0.15; 1 × 0.3; 2 × 0.45; 1 × 0.9	0.42	1 h; 2 h; 3 h; 6 h		+ total aliphatic glucosinolates, composition change in indolyl glucosinolates	
Strawberry	*Sphaerotheca aphanis*	Night	G	Panasonic Lighting Devices Co. UV-B lamp (peak at 280–315 nm)	0.864–2.03	0.08–0.188	3 h /daily 16×/11× or 4× fungicide application	+		^31^
					0.432–1.447 0.464–1.652	0.04–0.134 0.043–0.153	3 h + 16× fungicide application 3 h (4×/week) + 16× fungicide application	+ continuous UV-B treatment better		
*Catharanthus roseus*	*–*	Day	G	n.m. (solar)	4.84; 9.68; 14.53	1.345	1 h, 2 h, 3 h, followed by 72 h darkness	n.a.	1 h: + strictosidine, vindoline, catharanthine, ajmalicine 2 h: +strictosidine, vindoline, ajmalicine +/− catharanthine 3 h: + strictosidine, ajmalicine, +/− vindoline, - catharanthine	^135^
*Catharanthus roseus*	*–*	Day	L	UV illuminator *λ*_max_ 312 nm	0.028	0.23	2 min	n.a.	+ strictosidine, ajmalicine, catharanthine, tabersonine, serpentine, vindoline, vimblastine, 3’,4’-anhydrovinblastine + tryptophan decarboxylase	^136^
Broccoli	–	Day	G	Narrowband Philips TL F72T12 100 W/01	2.2; 8.8; 16.4	1.36; 2.29; 2.27	27 min, 64 min, 120 min	n.a.	+ phenolics & flavonoids -carotenoids, chlorophyll + sinigrin (glucosinolate) +/− glucotropaelolin	^35^
Broccoli	–	Day	L	TL 20 W/12 RS or TL 40 W/12 RS (Philips)	16.41; 24.01	2.28; 3.34	2 h	n.a.	+indolyl GS, aliphatic GS (low & high UV-B, 2 h and 24 h past irradiation, PI) Low UV-B: + total phenols (2 h past irradiation, PI), - total phenols (24 h PI) High UV-B: − total phenols (2 h past irradiation, PI); +/− total phenols (24 h PI) Change in individual compounds	^142^
Antarctic pearlwort	–	Day	L	Narrowband PL-L 36 W/01/4 P UV-B (Philips)	3.6; 21.6	0.05; 0.3	20 h	n.a.	+SA content +JA content (only high UV-B)	^145^
Barley	–	Day	L, WL	Narrowband TL 20 W/01 RS (Philips)	24.2	0.84	8 h	n.a.	+SA content (leaves and roots) +/− PAL activity	^154^
Tomato	–	Day	L	Narrowband TL 20 W/01 RS (Philips)	1.19	1.33	15 min	n.a.	Day 8: + gallic acid, catechin equivalents, SA content, +/− SAG (2-O-β-D-glucoside) Day 11: - Gallic acid, +/− catechin equivalents, - SA content, +SAG content Day 11+3 untreated: No differences	^155^
Arabidopsis	*–*	Day	L	Broadband TL 40 W/12 RS (Philips)	7.38	4.35	30 min	n.a.	Content depending on accession: + Ba-1, C24, T1080 Moderate: Col-0 +/− Lip-0, Mh-0	^157^
Arabidopsis	*–*	Day	L (sun simulator)	Sun simulator + cutoff filters in combination with high (1300 µmol m^−2^ s^−1^) and low (540 µmol m^−2^ s^−1^)PAR	6.05; 43.2	0.14; 1	12 h	n.a.	+ quercetin derivates	^166^
Soybean	Thrips (*Callosobruchus phaseoli)*	Day	Field	Solar control: UV-B blocking polyester films (cutoff below 310 nm)	n.m.	n.m.	n.m.	+/−	+quercetin and kaempferol triglycosides +/− SA content	^156^
Pepper	–	Day	L, WL	UV-XEFL 290BB (Ushio Lighting), 60% in 280–320 nm, 30 % in 320–400 nm, 4% in 200–280 nm, 6% 400–700 nm in combination with high (300 µmol m^−2^ s^−1^) and low (100 µmol m^−2^ s^−1^) PAR	4.98	1.38	1	n.a.	+ flavonol index for PAR300+UV, PAR300 vs. PAR100 or PAR100+UV	^165^
				In combination with (100 µmol m^−2^ s^−1^, either with 30% or 62 % blue light					+ total flavonoids and - anthocyanins for 30%Blue+UV and 62% Blue+UV	
Arabidopsis	*–*	Day (pulsed)	L	UV-B Narrowband TL 290–315 nm (Philips)	8.64	0.4	1 × 6 h 6 × 1 h (30 min recovery)	n.a.	+ flavonol content: sinapyl, kaempferol, quercetin (UV-B vs. control; pulsed vs. continuous) + *HY5, CHS, F3’H* (UV-B vs. control; partially pulsed vs. continuous)	^36^
Black Carrot	*–*	Day	L	Broadband TL40W/12 RS (Philips)	21.6	6	1 h	n.a.	+ anthocyanin content (only in roots) + total phenolics content (Roots + top) − JA content (top), +/− roots − SA content (top), +/− roots	^147^
Arabidopsis	–	Day	G	Philips TL12 tubes + cellulose acetate filters	7.7; 12.7; 86.4	1.07; 1.77; 6	2 h, 4 h	n.a.	+ changes in morphology, architecture + chlorophyll biosynthesis genes + specialized metabolites Stress induced only at the highest dose	^30^
Arabidopsis	*–*	Day	Field + L	Broadband TL40W/12 (Philips) +filters	6.3	7	15 min	n.a.	Transcriptome study	^94^

^a^*G* greenhouse, *L* laboratory/growth chamber, *WL* white light, *R* red, *B* blue. ^b^+: yes or higher; − no or reduced, +/− no difference. *n.m.* not mentioned, *n.a.* not applicable. Underlined values were calculated.

## UV-B effects, perception, and signal transduction

### UV-B light and its effects on plants

**Fig. 1:**
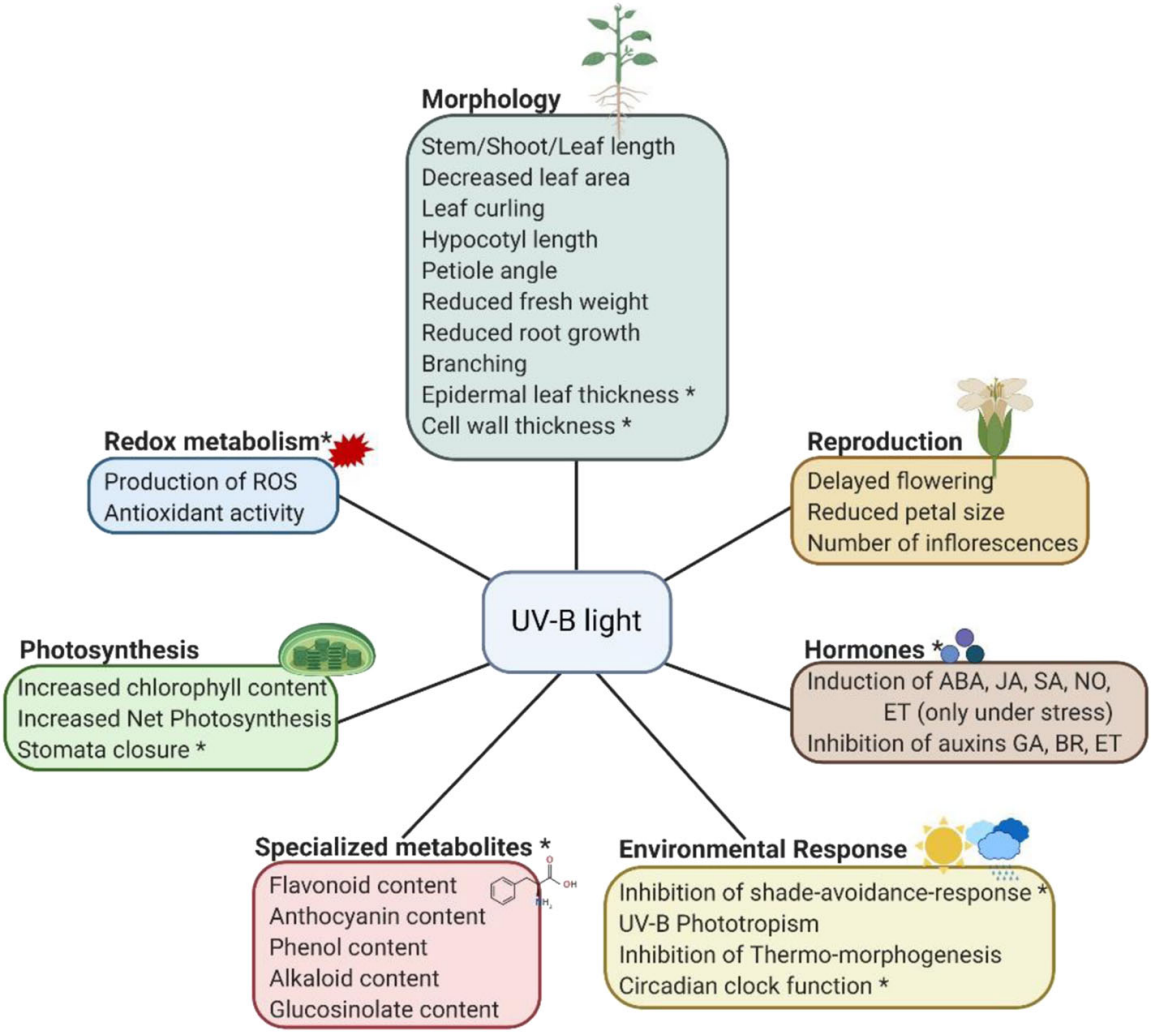
The effect of UV-B light on the plant. The asterisk marks a link with the defense response. ABA abscisic acid, JA jasmonic acid, SA salicylic acid, NO nitrogen oxide, ET ethylene, GA gibberellic acid, BR brassinosteroids

UV-B light influences several physiological, cellular and molecular responses (Fig. 1), including reproduction^26–31^, morphology^29,30,32–41^, photosynthesis^35,40,42–44^, specialized metabolites^34–36,40,42,45^, hormonal balances (reviewed in ref. ^46^), redox metabolism^47–49^, and environmental responses^29,50–52^. While UV-B irradiation mostly induces secondary metabolite biosynthesis, many plants show a bi-phasic response for morphological traits to different UV-B doses^53^. Meaning that UV-B can stimulate a specific response but can also repress this response, depending on the UV-B dose. This observation suggests that UV-B light can become a stress signal at a certain critical dose. Table 1 shows that these UV-B responses are very variable, depending on the plant species, accession, developmental stage (e.g., leaf number) perceiving UV-B radiation, as well as the experimental set-up (dose, intensity, timing, duration, source, and spectrum (narrowband UV-B lamps, broadband UV-B lamps or solar radiation) of UV-B light vs. greenhouse, field, or growth room studies)^13,53–55^.

### UV-B light signaling pathway

Arabidopsis has a single UV-B light receptor, the UVR8 photoreceptor, which is a 150 kDa protein, and occurs as an inactive homodimer in the cytoplasm. The UVR8 photoreceptor has been identified in many other plant species and crops including apple, tomato, grapevine, cucumber, and strawberry (reviewed in Tossi et al.^56^)^57,58^. UVR8 is constitutively expressed in all plant organs including leaves, roots, petals, and shoots^59–61^. The Arabidopsis *uvr8* mutant is deficient in UV-B-related photomorphogenesis and shows hypersensitivity to UV-B stress, which is manifested as stunted growth, leaf necrosis, and folding. The *uvr8* mutant also lacks the UV-B induced hypocotyl growth inhibition in response to UV-B irradiation^51,62,63^. In addition, Arabidopsis *uvr8* mutant shows a decreased accumulation of flavonoids in response to UV-B irradiation as well as a decrease in disease resistance to the fungus *Botrytis cinerea*^62,64^, but did not show altered resistance toward the herbivore *Spodoptera litura*^65^.

**Fig. 2:**
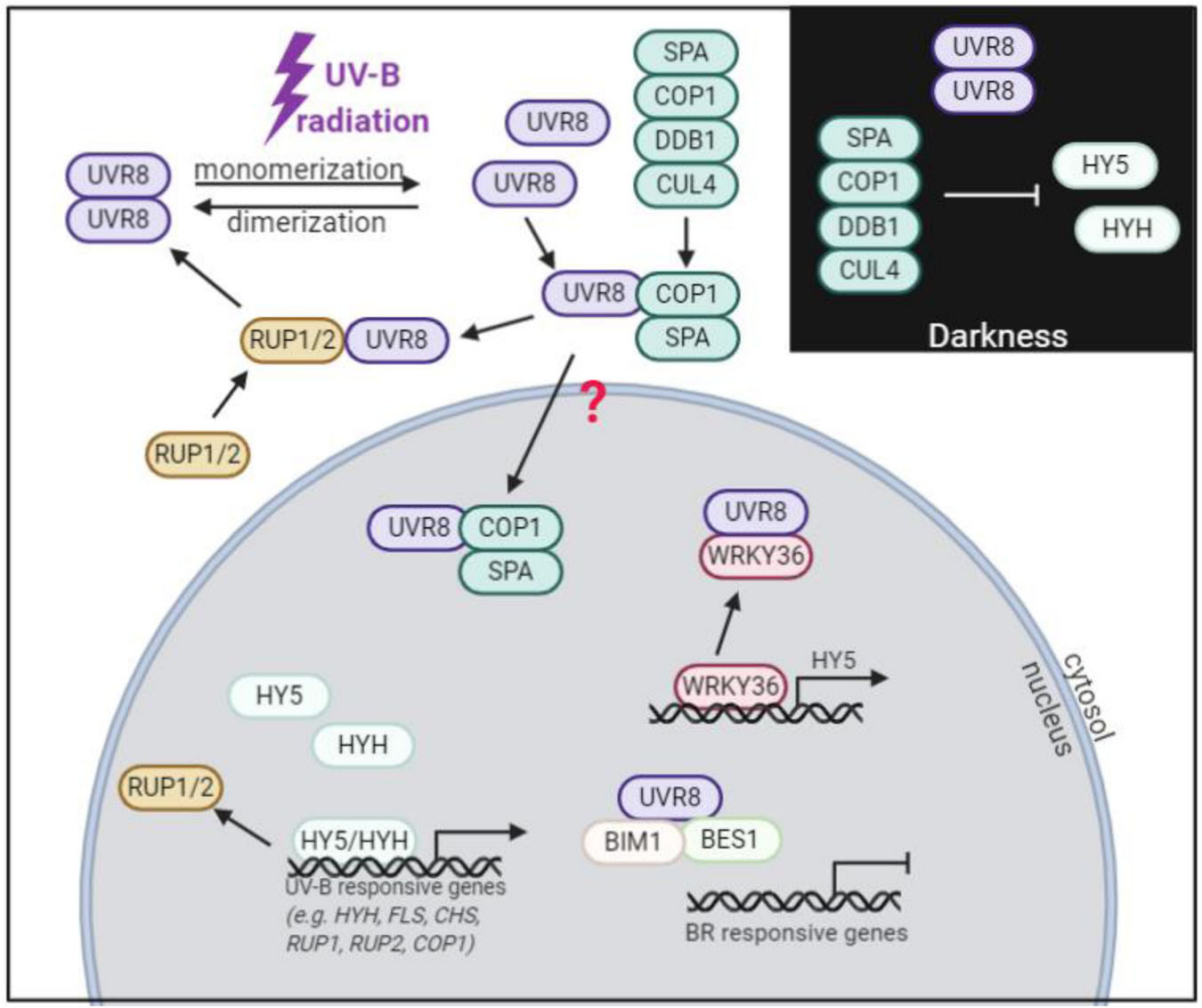
Schematic model of UV-B signaling. UVR8 monomerizes upon perceiving UV-B radiation. The COP1–SPA (CONSTITUTIVE PHOTOMORPHOGENIC 1; SUPPRESSOR OF PHYA-105) complex dissociates from the E3 ubiquitin ligase CUL4–DDB1 (CULLIN4; DNA DAMAGE BINDING PROTEIN 1) complex in the presence of UV-B radiation. UVR8 interacts with the COP1–SPA complex in its active monomeric form, which leads to a nuclear accumulation by an unknown process. UVR8 interacts with several transcription factors (TF) in the nucleus. Interaction with BIM1 (BES1-INTERACTING MYC-LIKE1) and BES1 (BRI1-EMS-SUPPRESSOR 1) leads to the inhibition of the transcription of brassinosteroid (BR)-responsive genes. Interaction with WRKY36 (WRKY DNA-BINDING PROTEIN 36) leads to the transcription of HY5 (ELONGATED HYPOCOTYL 5). The TF HY5 is a positive regulator of many UV-B responsive genes including HYH (HY5 HOMOLOG), FLS (FLAVONOL SYNTHASE), CHS (CHALCONE SYNTHASE), RUP1 (REPRESSOR OF UV-B PHOTOMORPHOGENESIS), RUP2, and COP1. The negative regulators RUP1 and RUP2 repress UVR8 function in the cytosol^60,61,67–75,77–79,85–90,175^

The UVR8 photoreceptor shows the structure of a seven-bladed ß-propeller and does not require any specific cofactor or chromophore to absorb UV-B light. Instead, UV-B light is perceived by closely packed tryptophan residues in the ß-propeller core and at the dimer interface residues^60,61^. UV-B light has no direct effect on the abundance of the UVR8 receptor but converts it from its inactive to its active form^59,60,66^. The inactive dimeric form is stabilized by cross-dimer salt bridges which are disrupted upon perceiving UV-B radiation, leading to the dissociation into two active monomers, which are subsequently translocated to the nucleus^60,61^. Only in its active monomeric form, UVR8 interacts with the COP1–SPA (CONSTITUTIVE PHOTOMORPHOGENIC 1; SUPPRESSOR OF PHYA-105) complex via a highly conserved amino acid sequence in the C-terminus (residues 397–423 of 440)^67,68^. The COP1–SPA E3 ligase complex is present in the nucleus in the dark and is negatively regulated by light, which induces its export to the cytosol, the disruption of the complex, and SPA degradation^69–71^. The COP1–SPA complex acts as an E3 ligation adaptor for CUL4–DDB1 (CULLIN4; DNA DAMAGE BINDING PROTEIN 1) to form an ubiquitination complex^71^. COP1–SPA alone or in combination with CUL4–DDB1 degrades transcription factors such as HY5 (ELONGATED HYPOCOTYL 5) and its homolog HYH (HY5 HOMOLOG), which are positive regulators of photomorphogenesis^72,73^. But in the presence of UV-B radiation, COP1–SPA dissociates from CUL4–DDB1, which allows COP1–SPA to interact with monomeric UVR8 (Fig. 2). Once COP1–SPA is bound to UVR8, UV-B light signaling is enabled by promoting HY5 and HYH stability and activity^73–75^. In contrast to the nuclear export of COP1 in response to white light, the interaction of COP1–SPA with UVR8 leads to a nuclear accumulation of the active monomer, although the mechanism behind this is not yet clear. The two recent models suggest a co-import of UVR8–COP1–SPA, mediated by the nuclear localization sequence of COP1 or the UVR8 monomer translocates to the nucleus, where COP1 inhibits its immediate export^76^. For further reading on the photoreceptor-mediated regulation of COP1–SPA, we refer to the review of Podolec & Ulm^71^.

In addition, UVR8 interacts with the transcription factor WKRY36 (WRKY DNA-BINDING PROTEIN 36), a repressor of *HY5* transcription, resulting in the transcription of *HY5* (reviewed in Liang et al.^76^) (Fig. 2)^77,78^. HY5, also called a master transcription factor due to its role in many different pathways, is involved in the expression of nearly one-third of the genes in Arabidopsis^79^. It does not only act downstream of the UV-B photoreceptor but it is also involved in blue, red, and far-red light signaling, mediated by the photoreceptors CRY1 (CRYPTOCHROME 1), PHYA (PHYTOCHROME A), and PHYB (PHYTOCHROME B)^80–84^. HY5 is involved in the regulation of light-inducible genes including e.g., *CHS (CHALCONE SYNTHASE)*, *MYB12 (MYB DOMAIN PROTEIN), RBCS-1A* (*RIBULOSE BISPHOSPHATE CARBOXYLASE SMALL CHAIN 1A*), *COP1*, *FHY1* (*FAR-RED ELONGATED HYPOCOTYL 1*), *HYH* (*HY5 HOMOLOG*), *CAB1* (*CHLOROPHYLL A/B BINDING PROTEIN 1*), *HFR1* (*LONG HYPOCOTYL IN FAR-RED*), *FHL* (*FHY1-LIKE*), *PKS1* (*PHYTOCHROME KINASE SUBSTRATE 1*), and *BBX22* (*B*-*BOX22*)^79,85,86^. Monomeric UVR8 also interacts and represses BES1 (BRI1-EMS-SUPPRESSOR 1) and BIM1 (BES1-INTERACTING MYC-LIKE1) transcription factors, which are activators of brassinosteroid response genes.

The same 27 amino acid sequence in the UVR8 C-terminus responsible for COP1 binding is also required for the binding of the negative regulators of monomerized UVR8 signaling, namely RUP1 and RUP2 (REPRESSOR OF UV-B PHOTOMORPHOGENESIS). The RUP proteins, present in the nucleus and cytosol, promote re-dimerization of UVR8 with 50% of the monomer re-dimerized after 20 min^87–89^. Although this binding can be achieved in the presence or absence of UV-B light, there are indications, that the interaction of UVR8 and RUP proteins is stronger in the presence of UV-B light (Fig. 2)^56,67,68,90^.

Importantly, UVR8 signaling depends on the activity of the UVR8 monomer and not on its total amount. Fast UVR8 monomerization and interaction with COP1 occur when plants grown in the absence of UV-B are suddenly exposed to UV-B radiation. Yet, an increase in UV-B radiation of UV-B-acclimated plants also results in higher levels of gene expression, e.g., of *HY5* without an increase in the UVR8 monomer. In UV-B-acclimated plants, binding of UVR8 to COP1 and RUP2 is only sustained minimally resulting in the basic level of gene expression, sufficient enough for UV-B acclimation. Exposure to higher levels of UV-B results in UVR8 dimer/monomer cycling by an increased association of UVR8 with COP1 but also RUP2 without a change in their total abundance^89^.

## High and low UV-B radiation: when does UV-B light become a stress signal?

UV-B radiation is often described as “low” or “high”, however, an exact definition for a low or high UV-B radiation intensity or dose is neither known nor at which intensity or dose it becomes a stress signal for the plant. Compared to visible light units, UV-B intensity is expressed in different units. Visible light intensity is often expressed in µmol m^−2^ s^−1^ while UV-B light intensity is expressed in W m^−2^. But more accurately, UV-B radiation is expressed in its daily dose (kJ m^−2^) by multiplying intensity (W m^−2^) with duration (in seconds). The unit conversion of W to kJ.s^−1^ (1 W = 0.001 kJ s^−1^) requires a multiplication with 0.001, according to Eq. (1):

1 $${{{{{\mathbf{UV}}}}}} - {{{{{\mathbf{B}}}}}}\,{{{{{\mathbf{daily}}}}}}\,{{{{{\mathbf{dose}}}}}}\,\left[ {{\mathrm{kJ}}.{\mathrm{m}}^{ - 2}} \right] = {\mathrm{intensity}}\left[ {{\mathrm{W}}.{\mathrm{m}}^{ - 2}} \right] \ast {\mathrm{duration}}\,\left[ {{\mathrm{sec}}} \right] \ast 0.001$$

**Fig. 3:**
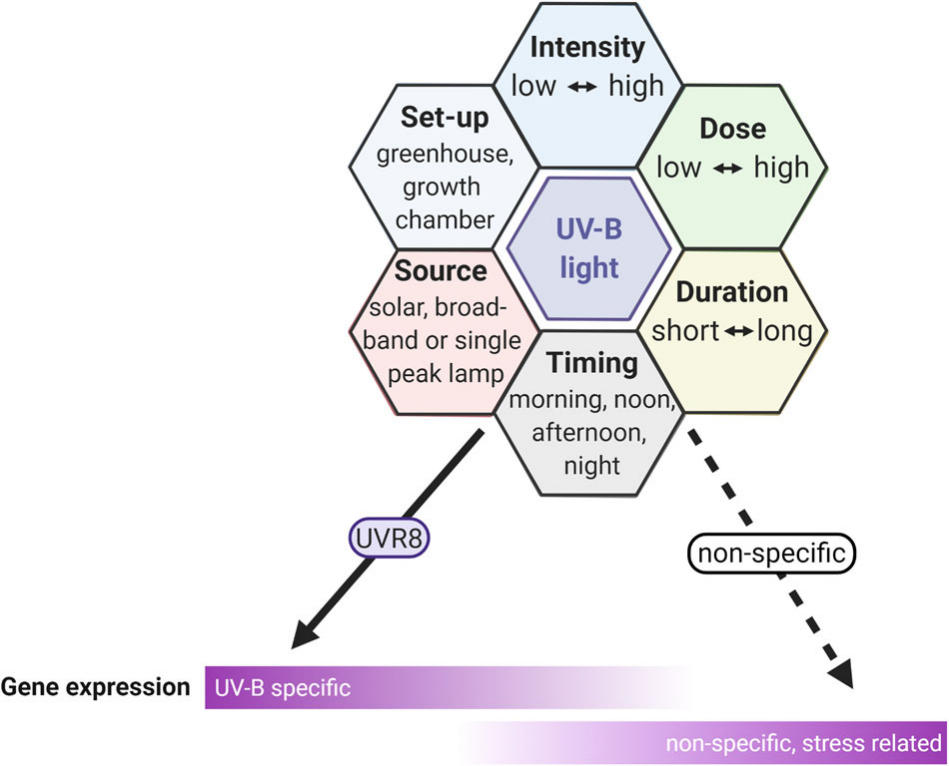
Variables affecting the perception of UV-B light by plants. UV-B light perceived by the UVR8 photoreceptor leads to the induction of UV-B-specific genes. UV-B radiation perceived as a stress signal induces nonspecific, stress-related genes. Some genes are inducible by both pathways.

There is species-specific and experimental set-up variation that makes it hard to define intensity or dose thresholds (Fig. 3), especially as UV-B treatments with the same daily dose but executed with UV-B lamps that have a different spectrum (narrow- vs broadband) can alter plant responses. A study in Arabidopsis showed that UV-B intensities of 1.07 W m^−2^ and 1.77 W m^−2^ (7.7 kJ m^−2^; 12.7 kJ m^−2^; 2 h) were labeled as non-stress inducing UV-B signaling^30^. These doses were able to induce UV-B light-dependent changes such as an altered morphology and architecture or “shown as”? (expressed as decreased rosette diameter and inflorescence height, and increased number of flowering stems), induction of chlorophyll biosynthesis genes, and accumulation of specialized metabolites, but no oxidative stress-related genes (ROS scavenging, ascorbate, and glutathione biosynthesis genes) were induced. A decrease in photosynthesis rate, which is a known stress response, was observed at a distinctively higher intensity of 6 W m^−2^ (86.4 kJ m^−2^; 4 h)^30^.

In general, both low and high UV-B radiation seem to trigger specific pathways dependent and independent of the UVR8 photoreceptor (Fig. 3)^81,91–94^. At low doses, UV-B light typically activates UVR8, which will lead to a diversity of changes, such as the alternation of flowering time, reduction of hypocotyl growth, UV-B-mediated phototropism, specialized metabolite biosynthesis, and epinasty. On the other hand, high UV-B radiation, especially given at a high intensity for a short time, can cause severe stress in plants. UV-B stress induces DNA damage and DNA repair mechanisms, reactive oxygen species (ROS) buildup with insufficient ROS scavenging capacity, MAPK kinase signaling, and wound/defense or general stress-related compounds (e.g., stress-related hormones, such as jasmonic acid, salicylic acid, and ethylene). Some of these stress responses are not only induced under high UV-B radiation but also upon other (a)biotic stresses^16,17,63,94–99^. Even low UV-B radiation can lead to increased ROS levels. However, at low UV-B levels, the plant is still able to oppose ROS with sufficient ROS scavenging^93,94,100^.

Common morphological and physiological changes induced by high UV-B stress include leaf tip burning, reduced leaf area and leaf elongation, an increase of leaf thickness, reduction in plant height, or reduced number of seed pods^30,51,53,101,102^. Besides morphological and physiological changes, several UV-B specific marker genes have been identified and assigned to different UV-B doses ranging from very low, low, intermediate to high^95,103^. However, there are differences between studies. For example, *MEB5.2* has been classified as a very low-level UV-B marker gene^103^, but it was also upregulated by high UV-B radiation (7 W m^−2^; 6.3 kJ m^−2^; 15 min) in Arabidopsis^94^. Moreover, *MEB5.2* was not differentially expressed in a study by Hectors et al.^30^ under UV-B radiation compared to non-exposed Arabidopsis, which emphasizes the difficulty of classifying a UV-B dose as “low” or “high”. Furthermore, it was shown that UV-B radiation below 1 µmol m^−2^ s^−1^ (= ~0.376 W m^−2^ for 313 nm) activates UVR8-dependent genes, while radiations above 1 µmol m^−2^ s^−1^ and up to 12 µmol m^−2^ s^−1^ (= ~4.512 W m^−2^ for 313 nm) induce the expression of both UVR8-dependent and -independent genes^81^. Some UVR8-dependent marker genes such as *CHS*, encoding a key enzyme in the flavonoid biosynthesis pathway, or the master transcription factor *HY5* appear to be inducible already at very low radiation of 0.1 µmol m^−2^ s^−1^ (= ~0.0376 W m^−2^ for 313 nm). Strikingly, it seems that low UV-B radiation does not result in fewer differentially expressed genes compared to high UV-B radiation in a full-genome transcriptome study^81,93^.

## UV-B triggers specialized metabolism to activate plant defense

The production of several specialized metabolites is activated upon UV-B perception by plants (Table 1). These metabolites play an important role in acclimation to UV-B radiation via direct screening (absorption of excessive UV-B) or via their antioxidative potential^104^. Many of these specialized metabolites are also induced by other stresses including biotic stress, where they have a role in plant-defense responses or ROS scavenging. During the past years mainly phenolic compounds (or polyphenols), but also alkaloids and glucosinolates, gained a lot of attention with regard to UV-B light acclimation and plant defense. Here, we provide a general overview on UV-B-induced specialized metabolites and the link with increased plant immunity.

### Phenolic compounds

**Fig. 4:**
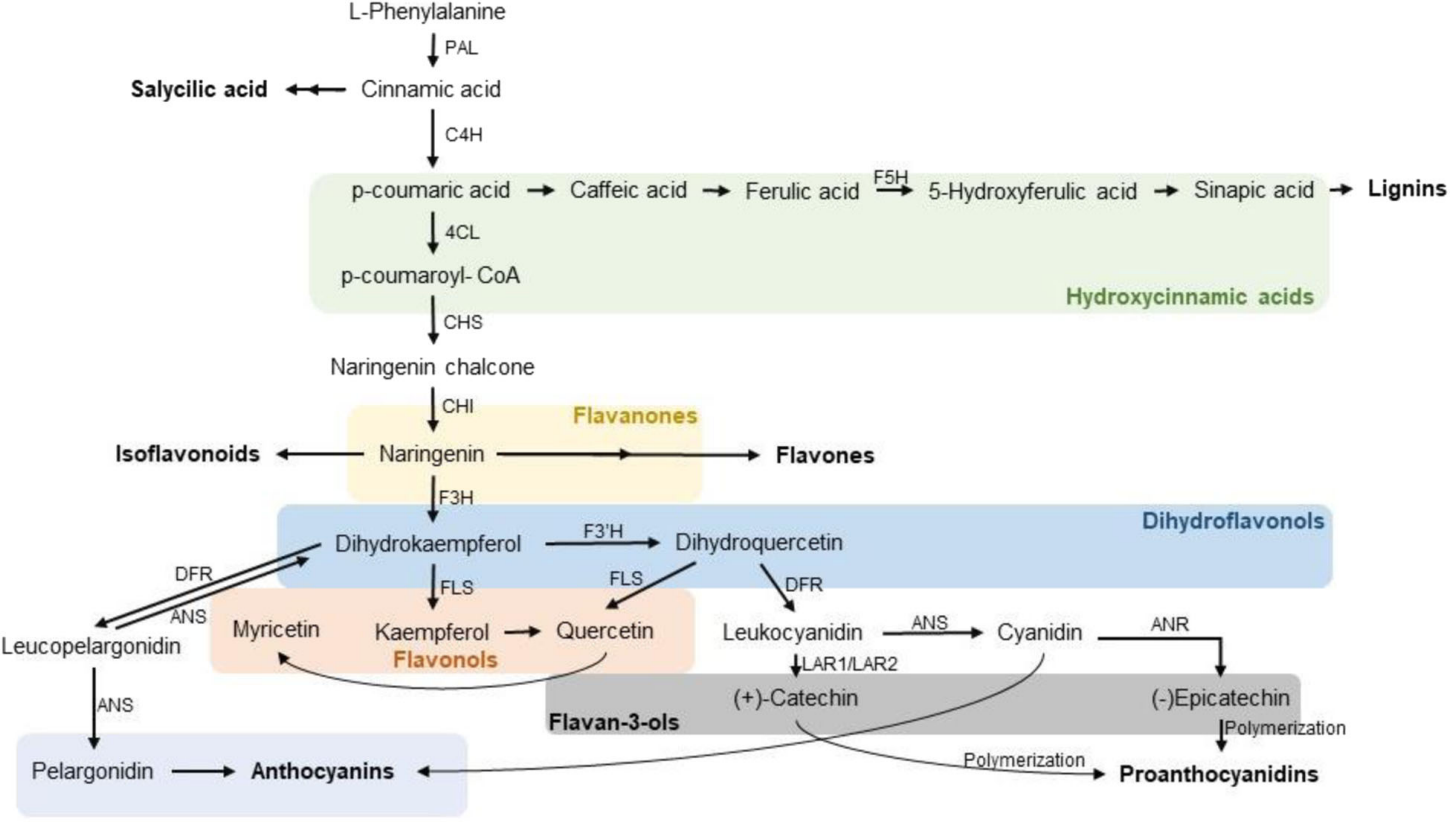
Simplified phenylpropanoid pathway including some main steps and enzymes. Colors represent different polyphenol groups. Green: hydroxycinnamic acids; yellow: flavanones; blue: dihydroflavonols; orange: flavonols; gray: flavan-3-ols; purple; anthocyanins. PAL PHENYLALANINE AMMONIA LYASE, C4H CINNAMIC ACID 4-HYDROXYLASE, 4CL 4-COUMARATE-CoA LIGASE, F5H FERULATE 5-HYDROXYLASE, CHS CHALCONE SYNTHASE, CHI CHALCONE ISOMERASE, F3H FLAVANONE 3-HYDROXYLASE, DFR DIHYDROFLAVONOL REDUCTASE, ANS ANTHOCYANIDIN SYNTHASE, FLS FLAVONOL SYNTHASE, ANR ANTHOCYANIDIN REDUCTASE

The phenylpropanoid pathway (Fig. 4) leads to the production of polyphenols, consisting of flavonoids, and non-flavonoids (e.g., hydroxycinnamic acids). Flavonoids include larger groups, including flavones, flavonols, flavan-3-ols, isoflavones, flavonones and (pro-) anthocyanidins, and smaller groups such as dihydrochalcones and dihydroflavonols^105^. Nonflavonoid compounds derived from hydroxycinnamic acids include chalcones, lignans, suberin, lignins, coumarins, and stilbenes^106,107^ (Fig. 4). Flavonoids and hydroxycinnamic acids are considered to be UV-light protectors due to their ability to scavenge ROS^108–110^. This is most probably why UV-B light mainly induces flavonoids with a higher hydroxylation level—such as quercetin derivatives—since flavonoids with multiple hydroxyl groups show a better ROS scavenging activity^110–112^.

Many biosynthesis genes of the phenylpropanoid pathway are UV-B light-inducible, including *PAL (PHENYLALANINE AMMONIA LYASE*), *C4H* (*CINNAMIC ACID 4-HYDROXYLASE*), *CHS*, *CHI (CHALCONE ISOMERASE*), *CHR* (*CHALCONE REDUCTASE*), *IFS* (*ISOFLAVONE SYNTHASE*), *F3H* (*FLAVANONE 3-HYDROXYLASE*), *F3’H* (*FLAVANONE 3’–HYDROXYLASE*), *DFR* (*DIHYDROFLAVONOL REDUCTASE*), *FLS* (*FLAVONOL SYNTHASE*), and *ANS* (*ANTHOCYANIDIN SYNTHASE*)^33,36,42,45,113–118^. These observations are corroborated by the reduced expression of several of these flavonoid biosynthesis genes (e.g., *F3H, CHS, CHI, DFR*) in the Arabidopsis *uvr8* and *hy5* mutants upon UV-B treatment compared to wild-type plants^92^. Concomitant, corresponding metabolites are inducible by low and high UV-B light in many plant species including rice, Arabidopsis, tobacco, soybean, and broccoli (Table 1)^65,113,119–123^.

It is important to note that some genes involved in the biosynthesis of the phenylpropanoid pathway are not continuously expressed upon supplemental UV-B radiation. In Arabidopsis, *CHS* expression has been shown to follow a dynamic expression profile during UV-B treatment: an increase shortly after the treatment, followed by a decrease back to its basal level. Also *F3’H* acts in a similar manner^36^. This underlines the importance of choosing accurate sampling time points when performing UV-B light studies.

Flavonoids and hydroxycinnamic acids are also upregulated in response to biotic stress^124–127^. Many phenolic compounds (e.g., flavones, genistein, nobiletin, and quercetin) show antifungal properties by altering the microbe cell wall permeability, interaction with fungal membrane proteins, or by directly inhibiting fungal growth in the apoplast (reviewed in Zaynab et al.^125^ and Arif et al.^128,129^). This might link UV-B light-induced specialized metabolites to improved disease resistance. For instance, increased resistance of Arabidopsis to the fungus *Botrytis cinerea* after irradiation with UV-B light was due to increased levels of sinapates (sinapoyl malate and sinapoyl glucose), derived from sinapic acid, a hydroxycinnamic acid. The increased level of sinapates was mediated by the UV-B photoreceptor UVR8 (see ref. ^64^). Similarly, UV-B triggered increased resistance to herbivores was accompanied by an increased amount of isoflavone glycosides in different soybean cultivars. The only cultivar nonresponsive to UV-B radiation and less resistant to pests showed no strong induction of isoflavonoids^119^.

### Alkaloids

Alkaloids are nitrogen-rich specialized metabolites found in ~20% of plant species^130^. More than 12,000 alkaloids have been described and they are divided into several classes, including terpenoid indole alkaloids, benzylisoquinoline alkaloids, tropane alkaloids, and purine alkaloids^130–132^. They are known for their role in plant-defense responses towards pathogens and many alkaloids are induced by low and high UV-B radiation in various plant species (Table 1)^104,125,133,134^.

The expression of several genes involved in alkaloid biosynthesis is reduced in the Arabidopsis *uvr8* and *hy5* mutants upon UV-B treatment compared to wild-type plants^92^. Many studies focused on the effect of UV-B radiation on terpenoid indole alkaloids. For example, in *Catharanthus roseus*, strictosidine, vindoline, catharanthine, tabersonine, and ajmalicine are induced in response to varying (low and high) doses of UV-B radiation^135,136^. This is supported by the activation of *TRYPTOPHAN DECARBOXYLASE*, a key enzyme in the terpenoid indole alkaloid biosynthesis, after irradiation of *C. roseus* leaves at a very low UV-B light dose and intensity^136^.

Camalexin (3-thiazol-2′-yl-indole), a broad-spectrum antimicrobial indole alkaloid phytoalexin present in Arabidopsis and a few other Brassicaceae species, is the most studied alkaloid in Arabidopsis. It is induced by bacteria, oomycetes, fungi, and viruses but also by UV-B radiation. Studies with Arabidopsis mutants defective in camalexin production confirmed that this alkaloid plays a major role in disease resistance against pathogens^132,137^. Camalexin holds the ability to damage bacterial and fungal cell membranes and can induce programmed cell death in *B. cinerea*^132^. While many Arabidopsis accessions produce camalexin, the accumulation in response to UV-B radiation and pathogens strongly depends on the accession tested^132,138^. This result indicates that caution is needed when transferring interpretations about UV-B-induced morphological and molecular changes from one plant species to another.

### Glucosinolates

Glucosinolates (GS), are sulfur-rich specialized metabolites, and consist of indolyl GS (derived from tryptophan), aromatic GS (derived from phenylalanine), and aliphatic GS (derived from methionine), and have been shown to play a role in plant resistance against insects and pathogens^132,139,140^. Glucosinolates are exclusively present in members of the Brassicales order. Intact GSs are stored in the vacuole and only show a limited biological activity, however, once the plant’s tissue is disrupted, endogenous myrosinases (a ß-thioglucosidase) hydrolyze the GSs into their biological active breakdown products. GS also accumulate under low and high UV-B radiation, but the response to UV-B radiation differs for each of the GS classes^141^.

For example, aliphatic GS of broccoli, especially methylsulfinylalkyl GS (3-methylsulfinylpropyl GS and 4-methylsulfinylbutyl GS), are inducible by both low and high UV-B doses, while indolyl GS are affected by prolonged exposure of several days of low UV-B radiation or a single high UV-B irradiation. The accumulation of aliphatic GS persisted even 3 days after a 5-day treatment, while the total indolyl GS content decreased back to its basal level, yet the profile of individual indolyl GS changed^35,141,142^. The aromatic GS glucotropaeolin only accumulates in response to high UV-B doses in broccoli florets^35^. Peculiarly, aliphatic GS do not accumulate in response to herbivory and the accumulation and profile of indolyl GS appear to be dependent on the individual insect but also differ from their UV-B profile in broccoli sprouts^142^.

Also the expression of genes involved in GS biosynthesis including *FMO GS-OX5* (*FLAVIN-MONOOXYGENASE GLUCOSINOLATE S-OXYGENASE 5*) and *CYP81F2 (CYTOCHROME P450, FAMILY 81, SUBFAMILY F, POLYPEPTIDE 2*) is inducible at low UV-B radiation while the expression of e.g., *2-ISOPROPYL-MALATE SYNTHASE 2* or *UPD-GLUCOSYL TRANSFERASE* decreases^141^.

While several studies showed induction of individual GS in response to UV-B light, Arabidopsis total GS content did not change after low UV-B light treatment^65^. However, it might be that individual compounds increased or decreased masking a change in total GS accumulation.

### Other metabolites induced by UV-B

#### Hormones

Many studies reported that a UV-B light treatment can influence the production of the plant hormones salicylic acid (SA) and jasmonic acid (JA). SA and JA are mainly involved in defense responses against biotrophic (SA) and necrotrophic pathogens and herbivory (JA), respectively^143,144^.

##### Jasmonic acid

UV-B light has been shown to induce JA production in many plant species including mung bean, rice, maize, tobacco, broccoli, Antarctic pearlwort, and Arabidopsis (reviewed in Vanhaelewyn et al.^46^)^17,65,141,145^. However, it has also been suggested that UV-B light can reduce JA and JA-Ile levels in e.g., UV-B-resistant soybean genotypes and black carrot^146,147^. Both high and low UV-B radiation have been shown to induce JA biosynthesis and JA signaling genes (reviewed in Vanhaelewyn et al.^46^, Jenkins^75^, and Ballaré^148^)^50,65^. However, the link between disease resistance, UV-B radiation, and JA is not yet fully clear, as several studies have reported contrasting results. The best examples of JA showing a positive effect on plant defense upon UV-B treatment are studies with insects. It appears that UV-B and herbivory similarly affect genes involved in defense response.

For example, LOX (lipoxygenase, part of the jasmonic acid biosynthesis pathway) activity increases in rice, and the jasmonic acid-responsive genes *PR-4* (*PATHOGENESIS-RELATED PROTEIN 4*) and *BG3* (*BETA-1,3-GLUCANASE 3*) are induced in broccoli after UV-B irradiation^113,114,141^. Moreover, UVR8 seems to be a key regulator of genes involved in JA biosynthesis (*AOS* (*ALLENE OXIDE SYNTHASE), AOC1* (*ALLENE OXIDE CYCLASE1), AOC3 (ALLENE OXIDE CYCLASE3) and OXOPHYTODIENOATE REDUCTASE3*), and signaling (transcription factor *WKRY70* and *JAZ1* (*JASMONATE ZIM DOMAIN1*)) in Arabidopsis under solar UV-B^149^. Interestingly, increased resistance of Arabidopsis to the herbivore *S. litura* after irradiation with a rather low UV-B dose for 5 days was due to increased JA levels, but independent of UVR8 (see ref. ^65^).

Several hypotheses have been formulated to explain the contradictory results in JA and JA-Ile accumulation in response to UV-B light. Besides varying experimental setups and the presence or absence of additional stresses, a buffering effect of wounding or herbivory-induced flavonoids on JA (and JA-Ile) signaling is considered. Meaning, a reduced accumulation of JA and JA-Ile might be caused by a higher flux through the flavonoid pathway. Thus, it has been suggested that certain flavonoids may act as general stress response modulators (reviewed in Ballaré^148^)^150^.

However, this is not supported by a recent study, where insect-fed and UV-B-treated plants showed a higher JA accumulation in Arabidopsis, maize, and tobacco compared to only UV-B-treated plants^65^. Alternatively, an increase in JA sensitivity, rather than JA accumulation, after a UV-B treatment was proposed to enhance plant defense. For instance, UV-B light leads to an increased expression of wound-responsive genes such as e.g., *TPI (TRYPSIN PROTEINASE INHIBITOR)*, without inducing JA accumulation in tobacco^151^.

The biosynthesis of specialized metabolites is often induced by JA because many JA-inducible transcription factors also trigger the expression of genes involved in the biosynthesis of glucosinolates, alkaloids, and phenylpropanoids^152^. However, many specialized metabolites are induced by UV-B light in JA biosynthesis mutants and it has been shown that the increased disease resistance of Arabidopsis to the fungus *B. cinerea* was not due to jasmonic acid signaling, but due to increased levels of sinapates, mediated by UVR8 (see refs. ^64,151^).

##### Salicylic acid

Several studies reported an effect of UV-B radiation on SA accumulation. However, comparable to JA, the link between UV-B, SA, and plant-defense responses are not always clear.

SA is derived from isochorismate, which is exported from the plastid to the cytosol via EDS5 (ENHANCED DISEASE SUSCEPTIBILITY 5). In the cytosol, PBS3 (avrPphB SUSCEPTIBLE 3) metabolizes isochorismate to isochorismate-9-glutamate. Spontaneous and non-enzymatic decomposition results in SA formation^153^. The enzyme PBS3 has been shown to be UV-B light-inducible in broccoli sprouts already at a low dose and intensity^141^. The production of SA in response to a low and high UV-B treatment has been demonstrated in different plant species, such as barley, Antarctic pearlwort, and tomato^145,154,155^. Interestingly, UV-B-irradiated tomato plants only showed a temporal increase of SA, but simultaneous an increase of the main inactive SA conjugate (SAG, 2-O-β-D-glucoside)^155^. In contrast to this, SA levels did not significantly increase in two soybean cultivars in response to solar UV-B radiation^156^. A similar observation was made in Arabidopsis, where only certain Arabidopsis accessions show SA accumulation in response to UV-B radiation in a genome-wide association study^157^. Thus, the difference in SA accumulation in response to UV-B light seems species-specific.

SA production is also linked with ROS accumulation, which is highly induced when plants perceive UV-B light stress. It was previously inferred that SA is mainly induced if the plant suffers from UV-B light stress or stress in general^46,149^ (reviewed in Vanhaelewyn et al.^46^). On the contrary, Arabidopsis radiated with UV-B light, which did not provoke signs of UV-B stress (no downregulation of genes involved in photosynthesis, no difference in chlorophyll content, no accumulation of hydrogen peroxide nor superoxide), showed an upregulation of SA and defense-related genes^121^. Another study on tomato plants perceiving 11 days of UV-B light showed no signs of UV-B stress, however, SA and SAG accumulated in these plants^155^.

##### Pathogenesis-related proteins

UV-B light can induce pathogenesis-related proteins in various plants^46,158,159^. These compounds include e.g., OLPs (OSMOTIN-LIKE PROTEIN), chitinases, PDF1.2 (PLANT DEFENSIN 1.2) and PR-1, -2, -4 and, -5 (PATHOGENESIS-RELATED PROTEIN)^17,101,111,114,158,160^. For instance, OLP, a PR protein with antifungal activity, is induced in response to UV-B radiation in rice and ß-1.3-glucanase (PR-2) is triggered by UV-B in both rice and strawberry^114,160^ and also in response to the pathogenic fungus *Magnaporthe oryzae*, causing rice blast^114^. However, *OLP* expression seems to be higher induced after a consecutive amount of days of UV-B radiation, while *ß-1,3-glucanase* is only highly expressed in the first day of UV-B radiation in strawberry^160^. Likewise, genes involved in fungal and bacterial pathogen defense of broccoli sprouts are inducible by UV-B radiation. This includes e.g., SA responsive genes *PR-1* and *PR-2* and SA and JA-responsive genes *PR-4* and *ß-1,3-glucanase*^141^.

We can conclude that low and high UV-B radiation stimulates the production of specialized metabolites including phenylpropanoids, alkaloids, and glucosinolates or their compositional change in various plant species. Their contribution to an increased disease resistance has been shown to be partially dependent and independent of the photoreceptor UVR8. While the effect of UV-B on the plant hormones JA and SA appears less clear, induction of PR proteins is generally observed. The degree of responses to UV-B treatment, however, seems to be species as well as treatment dependent.

## The effect of UV-B radiation on disease resistance in crops

Several studies focused on the use of supplemental UV-B light as a tool to increase disease resistance in various crops. A direct effect of UV-B light on the pathogen can never be excluded, as it has been shown that UV-B light affects fungi, beneficial microbes, or insect performance^65,161,162^. In this review, we mainly focus on the potential of UV-B light to reduce disease incidence through activating plant-defense mechanisms. In nearly all studies examined, a positive effect of UV-B on plant defense was observed. However, the different experimental setups make it challenging to compare the effect of UV-B radiation and draw general conclusions (Fig. 3 and Table 1). We have been able to group different UV-B studies based on their application method to reduce disease incidence in plants: (1) comparing the effect of different UV-B doses, (2) the effect of background radiation, (3) UV-B radiation during the night, and (4) UV-B priming and pulsed treatment.

### Increasing UV-B dose does not always correlate with increased disease resistance

There is a huge variation in the UV-B dose applied in different studies to increase disease resistance in plants. These daily doses can vary between 0.2 and 32 kJ m^−2^ (Table 1). It remains a challenge to uncover which dose is sufficient to induce a defense response, while not damaging the crop. For example, broccoli florets can show phototoxicity symptoms at a daily dose of 2.2 kJ m^−2^ (see ref. ^35^), while sunflower cotyledons can withstand daily doses of 30 kJ m^−2^ (see ref. ^163^). Depending on the UV-B dose, different pathways can be triggered, which either induce stress or a light acclimation response.

For instance, different doses of supplemental UV-B light were used to induce resistance of field-grown rice (*Oryza sativa* “Baijiaolaojing”) to rice blast caused by the fungus *Magnaporthe oryzae*^113^. In this study, all UV-B treatments (2.5, 5, 7.5 kJ m^−2^; 0.099; 0.198; 0.297 W m^−2^; 7 h; 280–315 nm) reduced rice blast disease incidence. However, a low and medium UV-B dose showed a more positive effect on disease resistance compared to the high dose, probably because plants treated with the higher dose suffered from UV-B stress. Concomitant, the amount of silicon, which often correlates with increased disease resistance against fungi, bacteria, and viruses (reviewed in Luyckx et al.^164^), was significantly enhanced for all UV-B treatments. Also, total flavonoids and phenols significantly increased under the medium and high UV-B dose, with the medium dose showing the highest value^113^.

In another study, higher doses of UV-B radiation (5–32 kJ m^−2^) were used to suppress powdery mildew (*Sphaerotheca aphanis* var. *aphanis*) infection in strawberry (*Fragaria x ananassa)*. All UV-B treatments performed equally well to suppress powdery mildew incidence. However, similar to rice, plants that perceived more than 8 kJ m^−2^ of UV-B radiation showed symptoms of phototoxicity^101^.

Another study tested the effect of 4 h and 6 h UV-B radiation (1–2 kJ m^−2^, 0.065–0.14 W m^−2^, 4 h or, 1.5–3 kJ m^−2^, 0.065–0.14 W m^−2^; 6 h; 265–385 nm) on disease susceptibility of greenhouse-grown roses (*Rose x hybrid* “Valerie” and “Rote Rose”) to powdery mildew (*Podosphaera pannosa*). Roses appear to be more sensitive to UV-B stress radiation compared to strawberry and rice, because both relatively low UV-B treatments induced leaf damage (visible leaf curling and sunscald). However, it has to be taken into account that the UV-B lamps of this study tailed into the UV-C region. In this study, 6 h of UV-B light completely suppressed powdery mildew infection in rose, while 4 h of UV-B light was only successful for one of the two rose cultivars^33^.

In Arabidopsis, several studies have been performed using relatively low (1 kJ m^−2^) to high (10 kJ m^−2^) UV-B doses to induce disease resistance to a variety of pathogens and pests. For example, 5 days of low UV-B radiation (1 kJ m^−2^; 0.318 W m^−2^; 3 × 17.4 min; 290–315 nm) induced resistance to the caterpillar *S. litura*^65^. An elevated resistance of Arabidopsis to the same caterpillar and additionally to the aphid *Myzus persicae* was also shown using UV-B radiation at a distinctively higher dose of 10 kJ m^−2^ (0.7 W m^−2^; 4 h; 290–315 nm). While plants did show smaller rosettes, it seems that the UV-B treatment did not lead to additional stress (no difference in chlorophyll content, ROS accumulation, nor reduced expression of photosynthesis-related genes), which is in contrast to strawberry, rice, and rose plants^121^. Likewise, Arabidopsis plants radiated with 5.5 kJ m^−2^ (0.382 W m^−2^; 4 h; 305–320 nm) UV-B light showed no visible damage or strong growth reductions, but an elevated disease resistance to the fungus *B. cinerea*^64^.

In conclusion, both low and high UV-B light doses can result in increased resistance to pests and pathogens in various plant species. Interestingly, it seems that low doses are always enough to obtain a positive effect. While high UV-B radiation appears to be safe in Arabidopsis, other plant species suffer from radiation stress already at lower doses, although it has to be taken into account that the UV-B lamp sometimes tails into the UV-C region. The dose when UV-B radiation causes stress symptoms is highly species-dependent.

### Background light during UV-B treatment alters its effects

Not only the intensity, duration, or dose of supplemental UV-B radiation is crucial to control disease incidence but also the background radiation and UV-B spectrum (narrow-peak UV-B lamp vs. broadband UV-B lamp vs. solar UV-B) play an important role^94,122,165,166^.

Supplemental end-of-the-day low UV-B radiation (0.3–0.9 kJ m^−2^; 1 W m^−2^; 5, 10, 15 min; 280–380 nm, peak at 313 nm), that was given after a 16-h light photoperiod (solar radiation supplemented with HPS lamps), suppressed powdery mildew (*Podosphaera xanthii*) incidence in cucumber (*Cucumis sativus* “Confida”)^122^. However, combining UV-B radiation with different background lights (16 h solar radiation supplemented with HPS lamps, followed by 10 min UV-B radiation (0.6 kJ m^−2^; 1 W m^−2^; 10 min) in the presence of an extra 2 h background light) altered this effect. Supplemental UV-A (15.84 kJ m^−2^; 2.2 W m^−2^) and blue light (39 µmol m^−2^ s^−1^) both suppressed disease symptoms but to a lesser extent than only a UV-B treatment (in the absence of other background light)^122^. The supplementary effect of blue and UV-B light was also studied in pepper plants (*Capsicum annuum L*.). The amount of blue light (30% or 62%) in a background PAR spectrum (100 µmol m^−2^ s^−1^) can have a positive effect on UV-B acclimation due to the production of specialized metabolites and less degradation of chlorophyll *a* and *b* and carotenoids^165^. These examples underline the importance of including information on the type of background radiation used in growth room studies.

Not only the background light quality but also the background light intensity influences the UV-B response. A high PAR light intensity (300 µmol m^−2^ s^−1^) resulted in a higher flavonol concentration in pepper plants compared to low PAR light (100 µmol m^−2^ s^−1^) with and without UV-B light (4.98 kJ m^−2^; 1.38 W m^−2^; 1 h; 280–400 nm). Interestingly, the high PAR intensity leads to a stronger accumulation of flavonoids compared to the low PAR in combination with UV-B light^165^. It would be interesting to investigate the effect of different light intensities on disease resistance further. It has been shown before, that high light intensities can facilitate the acclimation to higher UV-B doses by inducing a basic UV protection response making plants less susceptible to UV-B stress^102,166,167^. For instance, very high PAR (1310 µmol m^−2^ s^−1^) combined with very high (43.2 kJ m^−2^; 1 W m^−2^; 12 h) and medium (6.05 kJ m^−2^; 0.14 W m^−2^; 12 h) solar UV-B light induced more accumulation of quercetin derivatives compared to lower PAR (540 µmol m^−2^ s^−1^) combined with high UV-B intensities in Arabidopsis^166^. This is especially important when comparing studies conducted in indoor growth chambers, in greenhouses or outdoors. Equally important is the range of wavelengths of the UV-B source (solar vs. single peak vs. broadband). It has been demonstrated that lower UV-B wavelengths (295–290 nm) can have an antagonistic effect on the expression of genes normally induced by longer UV-B light wavelengths (305 nm)^94^.

### Low UV-B radiation at night might be more efficient than daytime applications

In contrast to daytime applications, it appears that night-time UV-B applications can already induce disease resistance at much lower intensities, durations, and doses. For example, a relatively low-dose UV-B treatment at night is sufficient to suppress symptoms of powdery mildew (*P. xanthii, P. aphanis, Golovinomyces biocellatus*) in cucumber (0.3–0.9 kJ m^−2^; 1 W m^−2^; 5, 10, 15 min; 280–380 nm, peak at 313 nm), strawberry and rosemary (*Salvia rosmarinus Rosy*) (0.288, 0.864 kJ m^−2^; 0.8, 1.6 W m^−2^; 6 min/day, 3 × 2 min/day or 18 min every 3rd day; 280–380 nm, peak at 313 nm)^122,168^. Interestingly, in cucumber, flavonol content decreased after the UV-B treatments in combination with inoculation of the pathogen compared to non-treated plants^122^.

Intriguingly, it has been shown in strawberry and rosemary that a UV-B irradiation every third night is sufficient to suppress disease severity with only minimal differences between varying intensities and durations while maintaining the same average daily dose for all treatments^168^. Low UV-B radiation at night was also able to reduce disease incidence in greenhouse-grown rose plants (*Rosa x hybrida*). Both a 2 h UV-B treatment at night (0.5–1 kJ m^−2^; 0.065–0.14 W m^−2^; 2 h; 265–385 nm) and a 6 h UV-B treatment at noon (1.5–3 kJ m^−2^; 0.065–0.14 W m^−2^; 6 h; 265–385 nm) were able to suppress powdery mildew (*P. pannosa*), but plants receiving 6 h of UV-B light showed signs of leaf damage^33^.

Another study aimed to use supplemental UV-B radiation at night to lower the standard amount of fungicide applications used in commercial greenhouse-grown strawberries against powdery mildew (*S. aphanis*). UV-B radiation (0.864–2.03 kJ m^−2^; 0.08–0.188 W m^−2^; 3 h; 280–315 nm) was given during the night for ~10 months and completely omitted powdery mildew infection for all plots receiving different amounts of fungicide applications^31^.

Interestingly, a continuous supplemental UV-B treatment at night (0.432–1.447 kJ m^−2^; 0.04–0.134 W m^−2^; 3 h) resulted in distinctively less disease incidence of greenhouse-grown strawberries exposed to powdery mildew, compared to an interrupted UV-B treatment (0.464–1.652 kJ m^−2^; 0.043–0.156 W m^−2^; 3 h, 4/week). The study of Suthaparan et al.^168^ contradicts this finding, as there were no notable differences in powdery mildew infection in strawberries between the interrupted and continuous treatment.

It is striking, that in all studies a rather low UV-B dose is sufficient to suppress disease symptoms if applied during the night. We can therefore conclude that UV-B applications during the night are more efficient than daytime applications.

A potential explanation why already very low doses of UV-B light at night are sufficient to suppress disease symptoms in different plant species might, in part, be due to a direct and more efficient effect on the pathogen during the dark. For example, the tolerance to UV-B radiation of the fungus *Colletotrichum acutatum* can be increased by visible light compared to radiation in the dark^169^. Moreover, the beneficial effect of UV radiation during the night on plant disease resistance has been previously observed in UV-C light studies^14,18^. It is presumed, that UV-C-light during a dark period inhibits the activation of the light-dependent DNA repair mechanism (photolyases) in some microorganisms^170^, which could also explain the strong effect of low-dose UV-B radiation at night. Indeed, the photolyase of the fungus *Pseudoidium neolycopersici*, the causal agent of powdery mildew in tomato, shows an action spectrum from 365 to 454 nm, indicating that the pathogen can recover from UV radiation damage if subsequently exposed to UV-A or blue light^171,172^. This is especially interesting as the spectrum of some UV-B lamps tail into the UV-C and UV-A region (265–385 nm), which can definitely influence the outcome of an experiment (Table 1). For instance, supplemental end-of-the-day UV-B radiation (0.3–0.9 kJ m^−2^; 1 W m^−2^; 5, 10, 15 min; 280–380 nm, peak at 313 nm), followed by darkness, was efficient to suppress powdery mildew (*Podosphaera xanthii*) incidence in cucumber. However, it showed to be less sufficient if followed by UV-A radiation or blue light^122^, probably due to activation of the pathogen’s photolyase. If the UV-B treatment was followed by red light, UV-B was equally efficient as if followed by darkness^122^.

### UV-B pulses and UV-B priming

Only a very limited number of studies investigated the effect of UV-B pulses or the effect of UV-B priming on plant-defense responses towards pathogens. Moreover, most of these studies focused on pests. It has been suggested that multiple short UV-B light treatments (pulses or sometimes referred to as interrupted irradiation) reduce the risk of UV-B damage, but can still increase disease resistance^65^. In a study of Qi et al.^65^, daily low UV-B doses were given in three pulses to tobacco, rice, maize, and Arabidopsis (Arabidopsis: 1 kJ m^2^; 0.318 W m^−2^; 3 × 17.4 min; tobacco, rice, maize: 3 kJ m^2^; 0.318 W m^−2^; 3 × 52.4 min; 290–315 nm) to increase resistance to *S. litura* (tobacco, Arabidopsis, maize) and *Mythimna separata* (rice). Even though the pulsed treatment increased resistance, it could not be concluded whether pulsing UV-B radiation leads to better results than continuous radiation, as a treatment with continuous UV-B radiation was not performed. Further, previous research showed that a similar dose of continuous UV-B radiation (1.5 kJ m^−2^; 0.0258 W m^−2^; 16 h) results in elevated resistance of Arabidopsis to *Lepidoptera* insects^173^. This could be an indication that pulsed UV-B treatment induces a similar effect as continuous UV-B radiation, showing that not the total duration, but maybe the total dose is a key factor in inducing disease resistance in plants. Besides disease resistance, a positive effect of pulsed UV-B radiation, compared to continuous high UV-B light (8.64 kJ m^−2^; 0.4 W m^−2^; 1 × 6 h or 6 × 1 h with 30-min recovery; 290–315 nm), was the stimulation of specialized metabolites in Arabidopsis^36^. Both UV-B treatments increased the amount of specialized metabolites including flavonols (kaempferol and quercetin) and sinapyl derivatives, but pulsed UV-B radiation (six doses with 30 min interval) showed a significantly higher accumulation compared to continuous UV-B light. However, both fresh weight and rosette diameter were negatively affected by both UV-B treatments, although this was more pronounced for continuous UV-B radiation^36^.

A possible explanation of the seemingly strong effect of the pulsed UV-B radiation could be an interference with the acclimation of plants to UV-B, constantly creating a response as if plants were non-acclimated. Continuous UV-B radiation leads to the acclimated state, where UV-B-responsive gene expression is maintained at a low level^89^. Surprisingly, according to the recent dynamic model of UVR8 signaling, it presumes that acclimated and non-acclimated plants react with a similar response, leading to a steady increase in downstream gene expression^89^. However, under pulsed UV-B radiation, the expression profile of *CHS, HY5*, and *F3’H* differs compared to continuous UV-B radiation in Arabidopsis, suggesting another mode of action between pulsed and continuous UV-B light^36^.

Most UV-B studies related to plant defense gave supplemental UV-B light prior to inoculation and the UV-B treatment lasted during the infection process. Studies rarely focused on comparing the priming/non-priming effect of UV-B treatment. Meaning, we have few clues about how long the UV-B effect persists to reduce disease susceptibility and we cannot rule out an additional effect of UV-B treatment on the pathogen or pest. For instance, glasshouse grown rice (‘Baijiaolaojing’) was irradiated (5 kJ m^−2^; 0.198 W m^−2^; 7 h; 280–320 nm) 3 days prior, during, and 3 days after inoculation with the fungus *M. oryzae*^114^. All treatments resulted in a significantly increased resistance to the rice blast fungus compared to the untreated control. However, priming plants 3 days prior to inoculation showed the highest increase in resistance. Concomitant, plants pre-treated with UV-B light before the inoculation showed significantly higher activity of PAL and CHT, a higher expression of *OsPAL* and *OsCHT* and a higher flavonoid and total phenol content compared to the UV-B treatment during and after inoculation. LOX activity was highest for the UV-B treatment during inoculation and ß−1,3-glucanase activity was similarly increased for the pre-treatment with UV-B and the irradiance during inoculation compared to UV-B irradiation after inoculation^114^. The positive effect of UV-B priming has also been demonstrated in broccoli sprouts, where UV-B (1 kJ m^−2^; 0.056 W m^−2^; 5 h; 280–360 nm) pre-treated plants were more resistant against aphids (*M. persicae)* and caterpillars (*Pieris brassicae)* compared to untreated plants^141^.

In conclusion, some studies suggest that pulsed UV-B treatment might affect downstream gene expression differently than continuous UV-B radiation and might reduce the risk of UV-B stress and damage. The positive effect of priming plants with UV-B prior to infection, suggests that UV-B radiation can act indirectly by increasing plant resistance and not only by affecting pathogens and pests directly. However, no information is available on how long these priming effects can last.

## Conclusion

UV-B radiation serves as a useful tool to reduce disease incidence in many different crops and in the model plant Arabidopsis. However, due to the large variability in the methods of applying UV-B light (dose, intensity, duration, timing during the day and night), and the species-specific and pathogen-specific responses, there is no single consensus UV-B response. The efficacy of a UV-B treatment is further complicated by the choice of the UV-B source and the background radiation.

We can conclude that UV-B light operates in two mechanisms (depending on the dose). Low doses of UV-B light can be beneficial to boost plant defense, however, higher doses can become disadvantageous as photo-oxidative stress radiation can cause growth retardation and morphological changes. The beneficial UV-B triggered plant defense is largely mediated by the UVR8 receptor and mainly stimulates the production of specialized metabolites, defense-related compounds, and often involves the plant hormones SA and JA to reduce disease incidence. The actual threshold when UV-B light becomes stress is highly species-dependent and varies depending on the spectral composition of the UV-B-lamps and the actual dose used. Furthermore, UV-B radiation followed by darkness appears to be more efficient than daytime applications, probably also due to the direct effect of UV-B light on the pathogen. Research on a pulsed UV-B treatment is scarce, but pulsed UV-B radiation could decrease the phototoxic effect. Furthermore, priming plants before an actual infection appears to be more efficient than using UV-B light as a direct tool to treat diseases or herbivory. Altogether, supplemental UV-B radiation has a huge potential beneficial effect on plant defense, but the multifaceted modes of application will determine the actual success or failure of a certain UV-B treatment in crop protection.
